# Discriminating mild traumatic brain injury using sparse dictionary learning of functional network dynamics

**DOI:** 10.1002/brb3.2414

**Published:** 2021-11-13

**Authors:** Liangwei Fan, Huaze Xu, Jianpo Su, Jian Qin, Kai Gao, Min Ou, Song Peng, Hui Shen, Na Li

**Affiliations:** ^1^ College of Intelligence Science and Technology National University of Defense Technology Changsha China; ^2^ Radiology Department Xiangya 3rd Hospital Central South University Changsha China

**Keywords:** dynamic functional connectivity, functional brain network, mild traumatic brain injury, sliding window analysis, sparse representation

## Abstract

Mild traumatic brain injury (mTBI) is usually caused by a bump, blow, or jolt to the head or penetrating head injury, and carries the risk of inducing cognitive disorders. However, identifying the biomarkers for the diagnosis of mTBI is challenging as evident abnormalities in brain anatomy are rarely found in patients with mTBI. In this study, we tested whether the alteration of functional network dynamics could be used as potential biomarkers to better diagnose mTBI. We propose a sparse dictionary learning framework to delineate spontaneous fluctuation of functional connectivity into the subject‐specific time‐varying evolution of a set of overlapping group‐level sparse connectivity components (SCCs) based on the resting‐state functional magnetic resonance imaging (fMRI) data from 31 mTBI patients in the early acute phase (<3 days postinjury) and 31 healthy controls (HCs). The identified SCCs were consistently distributed in the cohort of subjects without significant inter‐group differences in connectivity patterns. Nevertheless, subject‐specific temporal expression of these SCCs could be used to discriminate patients with mTBI from HCs with a classification accuracy of 74.2% (specificity 64.5% and sensitivity 83.9%) using leave‐one‐out cross‐validation. Taken together, our findings indicate neuroimaging biomarkers for mTBI individual diagnosis based on the temporal expression of SCCs underlying time‐resolved functional connectivity.

## Graphical abstract

We proposed a sparse dictionary learning framework to delineate spontaneous fluctuation of functional connectivity into the subject‐specific time‐varying evolvement of a set of overlapping group‐level sparse connectivity components (SCCs). The identified SCCs were consistently distributed in the cohort of subjects without significant inter‐group differences in connectivity patterns. Nevertheless, subject‐specific temporal expression of these SCCs could be used to effectively discriminate mTBI patients from healthy controls (HCs).

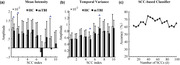

## 1 INTRODUCTION

The vast majority (70%−90%) of traumatic brain injury cases are mild and include those diagnosed as concussions (Cassidy et al., 2004; Roozenbeek et al., 2013). The actual number of mild traumatic brain injury (mTBI) cases may be much higher than those reported by hospitals due to generic symptoms in the early stage, such as headaches, dizziness, fatigue, and problems with concentration and memory (DeKosky et al., 2010; McCrea, 2008). Following the early acute stage (<3 days postinjury), mTBI may be associated with neurological symptoms and cognitive impairment that can impact an individual's quality of life (McAllister et al., 2006; R. Ruff, 2005). For example, a previous study found that patients with mTBI had worse visual tracking than healthy control subjects, which could lead to chronic disability (Maruta et al., 2010). Thus, it is critical to detect mTBI during the acute stage for guiding personalized interventions and treatment.

However, evaluating mTBI during the acute stage is challenging because existing mTBI diagnosis methods lack evidence of validity and reliability (Borg et al., 2004; R. M. Ruff et al., 2009; Vergara et al., 2017), especially for distinguishing mTBI from trivial head injury or moderate TBI (Kristman et al., 2014). Despite studies reporting that medical imaging methods—such as diffusion tensor imaging (DTI) and computed tomography (CT)—aid in diagnosing patients and evaluating injury severity (Huisman et al., 2004; Lipton et al., 2012), most patients with mTBI may not show obvious morphological abnormalities (Milman et al., 2005). Additionally, previous studies have shown that there are abnormal functional connectivities of mTBI patients in the prefrontal cortex (Slobounov et al., 2010), default mode network (DMN) (Sours et al., 2013; Zhou et al., 2012), sensorimotor network (SMN) (Vakhtin et al., 2013), and cerebellum (Nathan et al., 2015). Early abnormal findings on CT and magnetic resonance imaging (MRI) can improve long‐term clinical outcome predictions. For example, one study found clinical relevance of the early CT and MRI features to 3‐month outcomes in patients with mTBI (Yuh et al., 2013). Madhavan et al. (2019) and Palacios et al. (2017) demonstrated that functional connectivity at the semiacute stage (<3 weeks postinjury) could predict clinical outcomes after 3 weeks or later of injury. Therefore, it is vital to seek objective criteria for better detection of mTBI, especially in the early acute phase.

Recently, dynamic functional connectivity (dFC) based on functional magnetic resonance imaging (fMRI) has proven its effectiveness in the prediction and analysis of neuropsychiatric disorders, such as depression and schizophrenia (Damaraju et al., 2014; Liu et al., 2018; Qin, Chen, et al., 2015; Qin, Shen, et al., 2015; Su et al., 2016). Moreover, dFC can help characterize the aberrant interaction between functional brain networks, thus revealing the underlying disease pathology (Damaraju et al., 2014). Dynamic interactions across functional brain networks are related to behavioral and cognitive abilities (Hutchison et al., 2013; Qin, Chen, et al., 2015), and cognitive and behavioral impairment has been observed in patients with mTBI. Hence, we assumed that mTBI patients would exhibit abnormal dynamic interactions across functional brain networks, which could be used as potential neuroimaging biomarkers for individual diagnosis of mTBI.

Here, we propose a novel method to delineate the evolution of functional brain dynamic networks over time with time‐varying combinations of a set of overlapping sparse connectivity components (SCCs) based on dFC. This model is based on previous findings that a single region of the brain contributes to multiple functional networks (Lv et al., 2015; Shen et al., 2017) and that neural activity is not necessarily independent (Daubechies et al., 2009; Lee et al., 2011). The main idea of this method is to separate time‐resolved functional connectivities across brain networks into subject‐specific spatiotemporal structures that allow us to identify subtle postinjury abnormalities in these spatiotemporal structures as potential neuroimaging‐based biomarkers for individual diagnosis of mTBI. We demonstrate that the resulting SCCs reliably recur among subjects, without significant differences between mTBI patients and healthy controls (HCs). In addition, the temporal expression of these SCCs could discriminate mTBI patients from HCs. Therefore, the time‐varying combinations rather than the coupling profiles of SCCs are essential for revealing the pathologic basis of mTBI.

## 2 MATERIALS AND METHODS

This study was approved by the Institutional Review Board (IRB) of the Third Xiangya Hospital of Central South University and carried out in accordance with relevant Measures for the Ethical Review of Biomedical Research Involving Humans (Gursel, 2008). All study participants signed an informed consent form.

### 2.1 Participants

The dataset for this study included 31 mTBI patients and 31 HCs. The 31 mTBI patients (mean day postinjury = 2.1±0.8) were recruited in the emergency department of the Third Xiangya Hospital of Central South University. All the patients were included based on the criteria of the American Congress of Rehabilitation Medicine (Mayer et al., 2015; Medicine, 1993; Vergara et al., 2017). The specific inclusion and exclusion criteria could be found in our previous study (Shi et al., 2021).

Thirty‐one HCs matched on age, gender, and education were recruited from the surrounding community. More detailed demographic characteristics of participants are presented in Table 1. Moreover, each participant completed the behavioral and cognitive tests administered by a trained neuropsychologist, including the Wechsler adult intelligence scale test (WAIS‐IV) (Coalson et al., 2010), stroop test (Das, 2015), and the digit symbol test (Demakis et al., 2001). Resting‐state fMRI images were collected on a 3.0 T Philips MRI scanner with a 15‐channel head coil using an axial‐gradient spin‐echo sequence. Subjects were required to keep eyes closed and be awake during scanning. The parameters of data are as follows: repetition time/echo time (TR/TE) = 2000/30 ms, field of view (FOV) = 240 mm × 240 mm, flip angle (FA) = 90°, matrix size = 80 × 80, thickness = 3 mm, slices = 36, and volumes = 240.

**TABLE 1:** Demographics and clinical characteristics of participants

	mTBI patients (N = 31)	Healthy controls (N = 31)	*p*‐Value
Sex (M/F)	18/13	17/14	N/A
Age (year)	29.3 ± 5.2	33.1 ± 4.9	.58
Height (cm)	169.5 ± 10.1	170.1 ± 10.0	.41
Weight (kg)	70.4 ± 10.9	69.9 ± 9.2	.94
Education (year)	14.3 ± 3.1	13.0 ± 3.4	.12
Mechanism of injury	Traffic accident (22) Falling (6) Sport activity (3)	N/A	N/A
GCS score	13 (19) 14 (10) 15 (2)	N/A	N/A
LOC	<10 min (8) 10–20 min (18) 20–30 min (5)	N/A	N/A

Abbreviations: GCS, Glasgow coma scale; LOC; loss of consciousness; mTBI, mild traumatic brain injury.

### 2.2 Data preprocessing

The first five volumes of fMRI for each subject were removed because of magnetic saturation. The images then underwent motion correction, reslicing, and normalization to the Montreal Neurological Institute (MNI) space using SPM8 (http://www.fil.ion.ucl.ac.uk/spm), resulting in a voxel size of 3 mm × 3 mm × 3 mm. Next, the images were spatially smoothed using a 6‐mm full width at half maximum (FWHM) Gaussian kernel and temporally bandpass filtered from 0.01 to 0.08 Hz. Finally, we regressed the head movement, cerebrospinal fluid (CSF) signal, white matter (WM) signal, and their first‐order deviations to reduce spurious changes which were unlikely to be related to neural activity. In the current study, all 62 subjects had low mean head motion (Xu et al., 2018; <1 mm or 1°) during the scan and were included for the following analysis.

### 2.3 Time course extraction and dynamic functional connectivity estimation

A total of 160 regions of interest (ROIs) were used in this study (Dosenbach et al., 2010). Each ROI consisted of 27 voxels, including the centroid of the ROI and the 26 circumjacent voxels (radius = 6 mm). Briefly, the reliably activated voxels were identified based on meta‐analyses of fMRI activation data focused on memory, sensorimotor, language, default mode, and error‐processing functions. Then, for each meta‐analysis, peak‐finding algorithms were used for identifying the centroids of reliably activated groups of voxels. Finally, 160 ROIs were generated by combining the centroids and their circumjacent voxels in 6 mm diameter spheres. These ROIs cover much of the cerebral cortex. ROI‐based signals were generated by averaging voxel signals within each ROI. The voxels located outside the gray matter were excluded when calculating the ROI‐based signals. In addition, the 160 ROIs were grouped into six major functional networks, including the cerebellar (CB), cingulo‐opercular network (CON), DMN, fronto‐parietal network (FPN), occipital network (OCN), and SMN.

ROI time courses were generated by averaging BOLD signals of the center voxel and its neighboring 26 voxels. dFC was computed using a sliding‐window method with a window size of 60 s (30 TRs) and step size of 4 s (2 TRs), which was thought to achieve a balance between the ability to resolve the dynamics and quality of the covariance matrix estimation (Allen et al., 2012; Qin, Chen, et al., 2015). Specifically, the temporal correlations between all paired ROIs within each time window were calculated using Pearson's correlation coefficient, followed by Fisher's *r*‐to‐*z* transformation to achieve variance stabilization. Thus, a series of 160 × 160 correlation matrices were generated for each subject. The vectorized upper triangular part of each correlation matrix was extracted for further analysis. Consequently, for each subject, we obtained a sliding‐window correlation matrix $S^{\left(i\right)}\in R^{w\times p}$, where i=1,2,…,m is the index of subjects, w is the number of windows, and p=160(160−1)/2 is the number of node pairs.

### 2.4 Identification of SCCs

**FIGURE 1:**
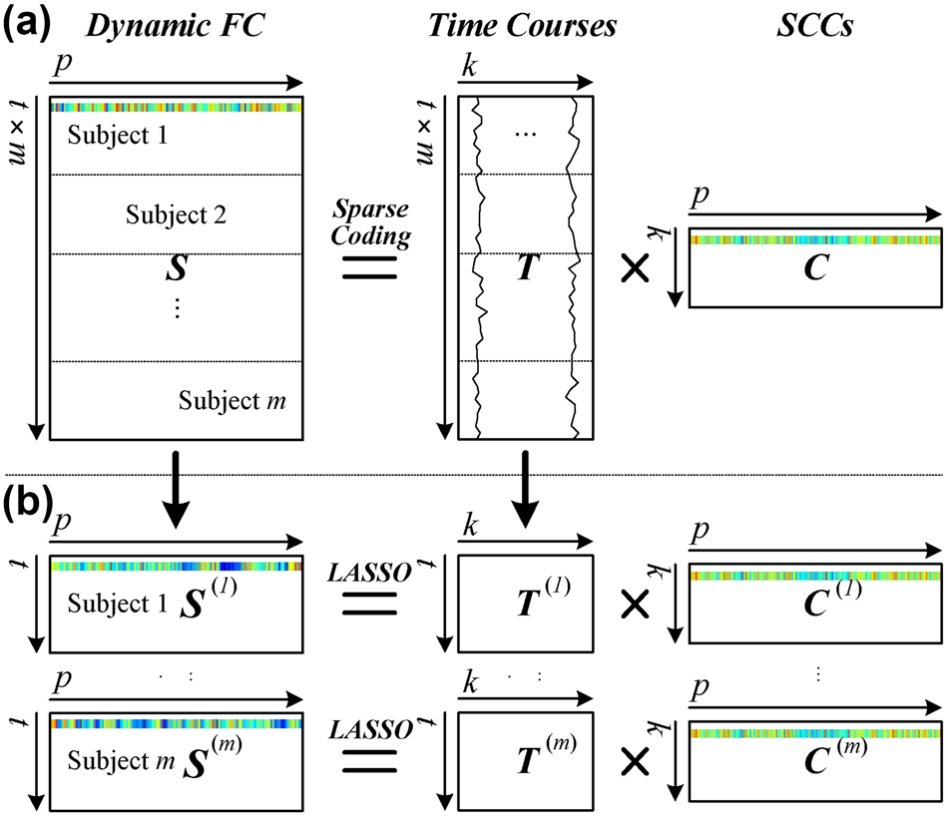
The process of decomposing resting‐state dynamic functional connectivity into sparse connectivity components (SCCs). (a) The group‐level sparse representation and (b) the subject‐specific sparse representation

A schematic diagram illustrating our sparse learning approach is depicted in Figure 1. First, we combined the sliding‐window correlation matrices derived from all subjects into one matrix $S\;=\;\left[S^{\left(1\right)};S^{\left(2\right)};\ldots ;S^{\left(m\right)}\right]\in R^{t\times p}$, where t=w×m is the total number of windows for all subjects. Then, we used a generic formulation of matrix factorizations to decompose the concatenated structure into a fixed number of common SCCs as follows (Figure 1a):

(1) S=T×C.

The concatenated sliding‐window correlation matrix *S* can be represented as the multiplication of *k* common SCCs, stored as the rows of the matrix $C\;=\;\left[c_{1:};c_{2:};\ldots ;c_{k:}\right]\in R^{k\times p}$, and their associated time‐dependent weights in $T\;=\;\left[t_{:1},t_{:2},\ldots ,t_{:k}\right]\in R^{t\times k}$. Each column in *T* is the time course of the corresponding common SCC. Computing the row norms of *C* allowed us to rank the common SCCs according to their importance for decomposition.

Equation (1) aims to solve an L1‐regularized LASSO problem:

(2) $$\min_{T\in R^{t\times k},C\in R^{k\times p}}\frac{1}{2}{∥S-TC∥}_{2}^{2}+\lambda ∥\alpha {∥}_{1},$$

where *λ* is a parameter for a regression residual and sparsity level trade‐off. In addition, each column of *T* is constrained by Equation (3) to avoid a trivial solution of the optimization.

(3) DΔ=T∈Rt×k,s.t.∀j=1,…,k,t:jTt:j≤1.

The Sparse Modeling Software toolbox of MATLAB (SPAMS, http://spams‐devel.gforge.inria.fr/) was used to find the optimal solution in Equation (2) by alternately updating either matrix *T* or *C* while the other was held constant (Mairal et al., 2009).

Next, we split the common temporal matrix *T* into *m* subsets, each of which corresponds to one subject. Therefore, Equation (1) can be rewritten as follows:

(4) $$\left[\begin{array}{c} S^{\left(i\right)}\\ \vdots \\ S^{\left(m\right)} \end{array}\right]=\left[\begin{array}{c} T^{\left(i\right)}\\ \vdots \\ T^{\left(m\right)} \end{array}\right]\;\times C,$$

where $S^{\left(i\right)}$ represents the sliding‐window correlation matrix of the *i*th subject, and $T^{\left(i\right)}\in R^{w\times k}$ is the subject‐specific temporal matrix corresponding to the *i*th subject. Thus, for the subject *i*, the subject‐specific SCCs $C^{\left(i\right)}\in R^{k\times p}$ can be obtained by resolving an L1‐regularized LASSO problem as follows:

(5) $$\min_{C^{\left(i\right)}\in R^{k\times p}}\frac{1}{2}∥S^{\left(i\right)}-T^{\left(i\right)}C^{\left(i\right)}{∥}_{2}^{2}+\lambda ∥C^{\left(i\right)}{∥}_{1}.$$

This would produce the projection of individual data $S^{\left(i\right)}$ on the subject‐specific SCCs $C^{\left(i\right)}$ and the corresponding temporal matrix $T^{\left(i\right)}$. Thus, we can reconstruct the individual data $S^{\left(i\right)}$ as follows (Figure 1b):

(6) $$S^{\left(i\right)}=T^{\left(i\right)}\;\times C^{\left(i\right)}.$$

We also used SPAMS to find the optimal solution in Equation (5) by updating $C^{\left(i\right)}$ while $T^{\left(i\right)}$ was held constant.

To evaluate the significance of each SCC, we first identified the subject‐specific SCCs with the corresponding time‐varying weights for each subject. Then, a one‐sample *t*‐test (*p *< .05, false discovery rate [FDR] correction) with the null hypothesis of disconnection was conducted on each subject‐specific SCC across subjects. Connectivity that did not survive the test was set as zero. The resulting *t*‐maps were regarded as the group‐level SCCs. In addition, we also detected the potential changes in the coupling strength of SCCs between mTBI patients and HCs by performing a two‐sample *t*‐test on each SCC across subjects.

We reconstructed the network dynamics via multiplying the learned common SCCs with their associated time‐dependent weights, and the potential effects of this reconstruction were explored from a frequency‐domain view. Specifically, the residual correlation time series was obtained by subtracting the reconstructed correlation time series from the original correlation time series for each subject. Then, the fast Fourier transform (FFT) was applied to the original, reconstructed, and residual correlation time series to evaluate the spectral distributions of each connection pair.

### 2.5 Parameter selection

The free parameters of this model are the number of SCCs *k* and the sparsity level of each SCC *λ*, which may affect the model's performance of fitting the observed data. In particular, the approximation error declines when *k* is increased or *λ* is reduced. However, this may result in over‐fitting beyond a certain value of *k* or *λ*. A grid search based on a twofold cross‐validation measure was performed to find the optimal parameters. First, for each pair of *k* and *λ*, we calculated the matrix of the common SCCs $C_{\mathrm{train}}$ on the training dataset as given in Equation (2). Then, for each subject in the testing dataset, we calculated the subject‐specific temporal matrix as follows:

(7) $$T_{\mathrm{test}}^{\left(i\right)}=S_{\mathrm{test}}^{\left(i\right)}\;\times C_{\mathrm{train}}^{T},$$

where $i\;=\;1,2,\ldots ,m^{\mathrm{test}}$ and $m^{\mathrm{test}}$ is the number of subjects in the testing dataset. The subject‐specific SCCs $C_{\mathrm{test}}^{\left(i\right)}$ for each subject in the testing dataset can then be obtained as given in Equation (5). Finally, the cross‐validation measure of the error was computed on the testing dataset relative to the variance in the test data as follows:

(8) $$\text{Test}\;\text{error}\;=\frac{\sum_{i=1}^{m^{\mathrm{test}}}{∥S_{\mathrm{test}}^{\left(i\right)}-T_{\mathrm{test}}^{\left(i\right)}\times C_{\mathrm{test}}^{\left(i\right)}∥}_{2}^{2}}{\sum_{i=1}^{m^{\mathrm{test}}}{∥S_{\mathrm{test}}^{\left(i\right)}-{\bar{S}}_{\mathrm{test}}∥}_{2}^{2}},$$

where ${\bar{S}}_{\mathrm{test}}$ is the subject‐average correlation matrix of the testing dataset. The values of *k* and *λ* at which the error does not drop significantly are chosen as the operating point.

### 2.6 Classification of mTBI using temporal features of SCCs

To test the capacity of our method for classification, the support vector machine (SVM) classifier was employed for discrimination between mTBI patients and HCs based on the temporal features of SCCs (mean intensity and temporal variance of time courses) with leave‐one‐out cross‐validation. In each cross‐validation run, the *k* common SCCs were first identified based on the training dataset. Then, the subject‐specific temporal matrix corresponding to the SCCs was calculated for each subject in the training and testing datasets as given in Equation (5). The mean intensity and temporal variance of the time course of SCCs were calculated and concatenated as temporal features (2 × *k* dimension) of SCCs for each subject. Finally, a linear SVM classifier was trained on the training dataset and applied to the testing samples. We performed the classification analysis with different values of *k* to find the optimal point with the highest classification accuracy.

### 2.7 Reproducibility and control analysis

To assess the reproducibility of the SCCs across subsamples, we first randomly divided the 62 subjects mentioned above into three groups. Then, the sparse dictionary learning approach was repeated to capture the SCCs for each group. The SCCs derived from the groups and those from the full sample were further compared. To determine whether the SCCs critically depended on the window or step size in the sliding‐window analysis, we repeated the analysis with different window sizes (30−120 s with a step of 10 s) and step sizes (1−20 TRs with a step of 2 TRs) on the full sample. The SCCs derived with different parameters were then compared with the SCCs derived with the window size of 30 s and step size of 1 TR. Herein, we used the $\eta^{2}$ to estimate the similarity of two SCCs as follows:

(9) $$\eta^{2}=\;1-\frac{\sum_{i\;=\;1}^{p}\left[{\left(a_{i}-m_{i}\right)}^{2}+{\left(b_{i}-m_{i}\right)}^{2}\right]}{\sum_{i\;=\;1}^{p}\left[{\left(a_{i}-\bar{M}\right)}^{2}+{\left(b_{i}-\bar{M}\right)}^{2}\right]},$$

where $a_{i}$ and $b_{i}$ are the values in position *i* of SCC *a* and SCC *b*, $m_{i}$ is the mean of $a_{i}$ and $b_{i}$, and $\bar{M}$ is the grand mean value across all positions in the two vectors.

## 3 RESULTS

### 3.1 Clinical results

As shown in Table 1, there were no significant differences (*p* > .10) between the mTBI patients and HCs on the demographics, which indicated that the participants were matched. The behavior statistics and two‐sample *t*‐test results for mTBI patients and HCs are summarized in Table 2. We found a significant difference between mTBI patients and HCs for only the digit symbol task (*p* < .05).

**TABLE 2:** Behavioral measures and two‐sample *t*‐test analysis results (*p < .05)

		Mean ± SD		
Index	Metrics	mTBI patients	Healthy controls	*p*‐Value
1	WAIS‐IV	102.80 ± 13.36	97.94 ± 13.28	.159
2	Digit symbol	54.70 ± 9.81	62.29 ± 13.41	.015*
3	Stroop_W	24.21 ± 5.78	22.22 ± 7.28	.242
4	Stroop_C	36.99 ± 5.34	37.25 ± 11.85	.913
5	Stroop_D	61.14 ± 14.27	64.33 ± 22.87	.517
6	Stroop_F	1.20 ± 1.42	1.74 ± 2.16	.254
7	Stroop_D‐C	24.15 ± 12.07	27.08 ± 14.65	.397

Abbreviations: mTBI, mild traumatic brain injury; WAIS‐ IV, Wechsler adult intelligence scale test.

### 3.2 Group‐level SCCs underlying functional network dynamics

**FIGURE 2:**
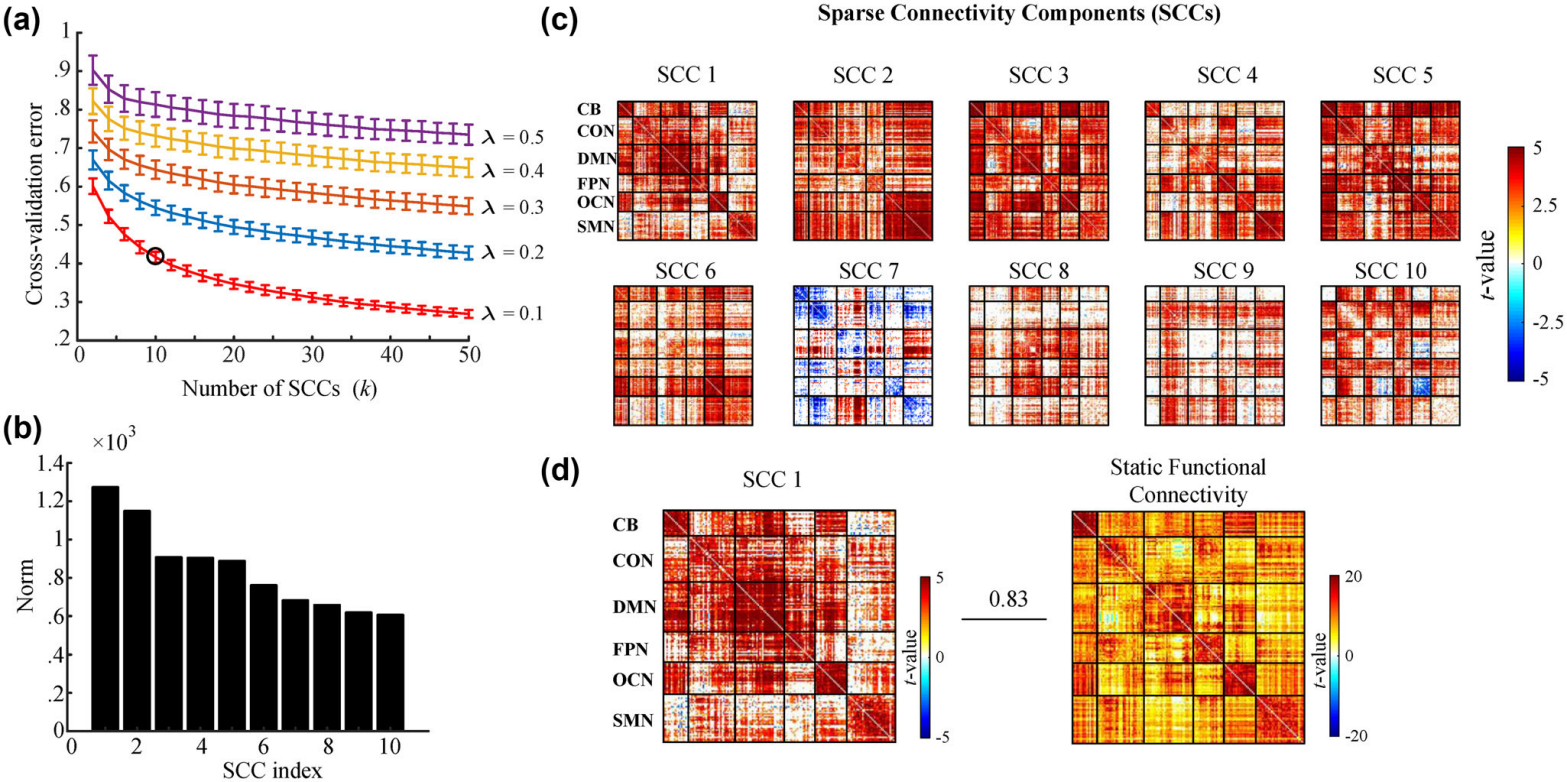
Extracting the group‐level sparse connectivity components (SCCs) of resting‐state functional network dynamics. (a) Grid search results for parameter selection. (b) The SCCs were ranked according to their importance for decomposition by their row norm. (c) The correlation maps of SCCs. (d) SCC 1 exhibits a highly similar connectivity profile to the static connectivity map ($\eta^{2}=\;0.83$)

The output of the cross‐validation for parameter selection is illustrated in Figure 2a. The cross‐validation error saturated beyond λ=0.1, and a clear elbow point of the cross‐validation was observed beyond k=10, indicating that a number of SCCs above 10 can sufficiently fit the observed data. Thus, the point of k=10 and λ=0.1 was chosen as the operating point.

Consequently, 10 group‐level SCCs were obtained for all participants. These SCCs were ranked according to their importance for decomposition by calculating the row norm of each SCC (Figure 2b). The *t*‐value maps (one‐sample t‐test, *p* < .05, FDR‐corrected) of the resulting 10 SCCs are shown in Figure 2c. These SCCs exhibited structuralized correlation patterns.

**FIGURE 3:**
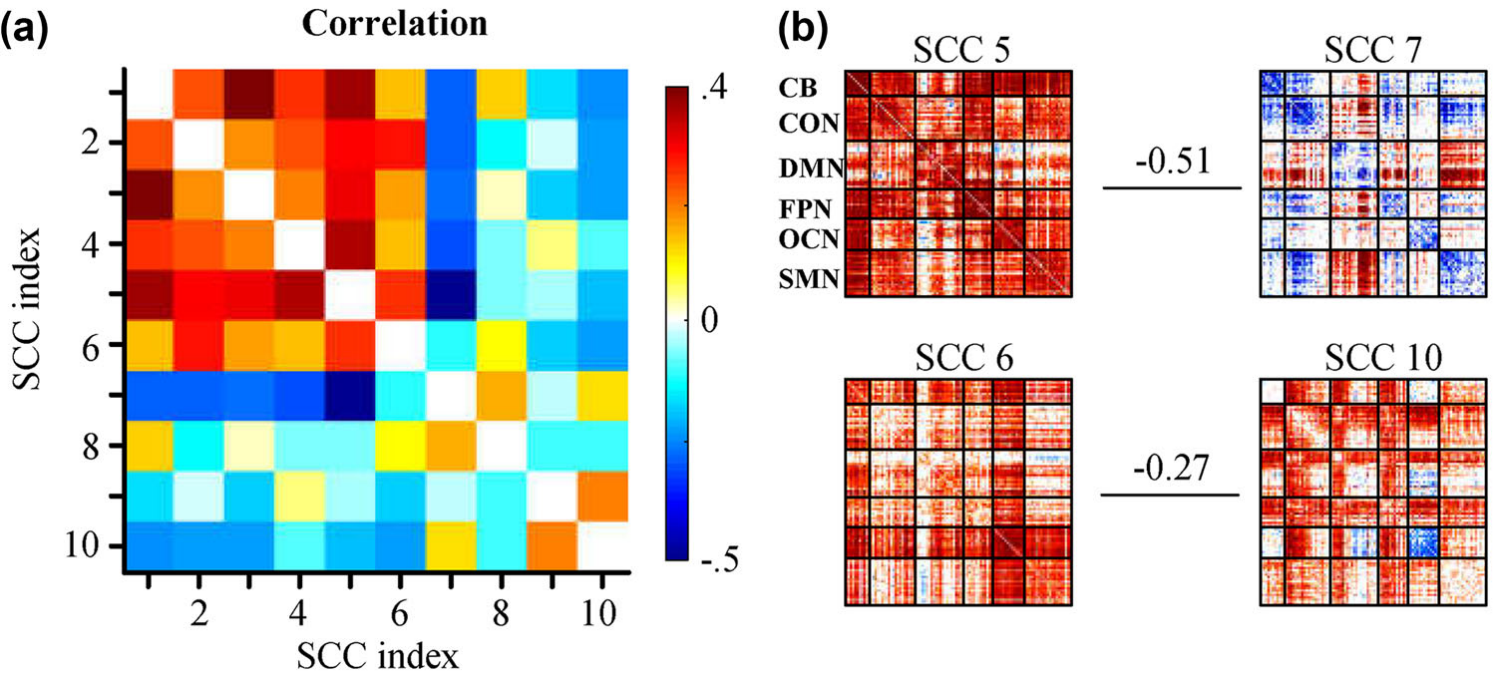
(a) The spatial similarity map across the sparse connectivity components (SCCs) was calculated using Pearson's correlation coefficient. (b) The strong negative correlations of SCC pairs

Among these 10 SCCs, SCC 1 is notable due to its predominantly strong positive correlation within the six functional networks. More importantly, SCC 1 showed a highly similar connectivity profile to the static connectivity maps ( $\eta^{2}=\;0.83$, Figure 2d). The spatial similarities across SCCs were further evaluated using Pearson's correlation coefficient. Notably, significant spatial correlation or anticorrelation was observed in some pairs across the low‐index SCCs. In contrast, high‐index SCCs exhibited relatively weak spatial similarity partially due to the increased sparsity of connectivity within these SCCs (Figure 3a). Intriguingly, strong negative correlations were found in the SCC 5–SCC 7 and SCC 6–SCC 10 pairs (Figure 3b). An anticorrelation between the DMN regions and task‐positive systems was observed within the SCC 5–SCC 7 pair. This anticorrelation has been suggested to index moderately stable individual behavioral differences (Kelly et al., 2008) and is linked to individual attention level (Thompson et al., 2013).

### 3.3 SCCs temporally disengage functional network dynamics

**FIGURE 4:**
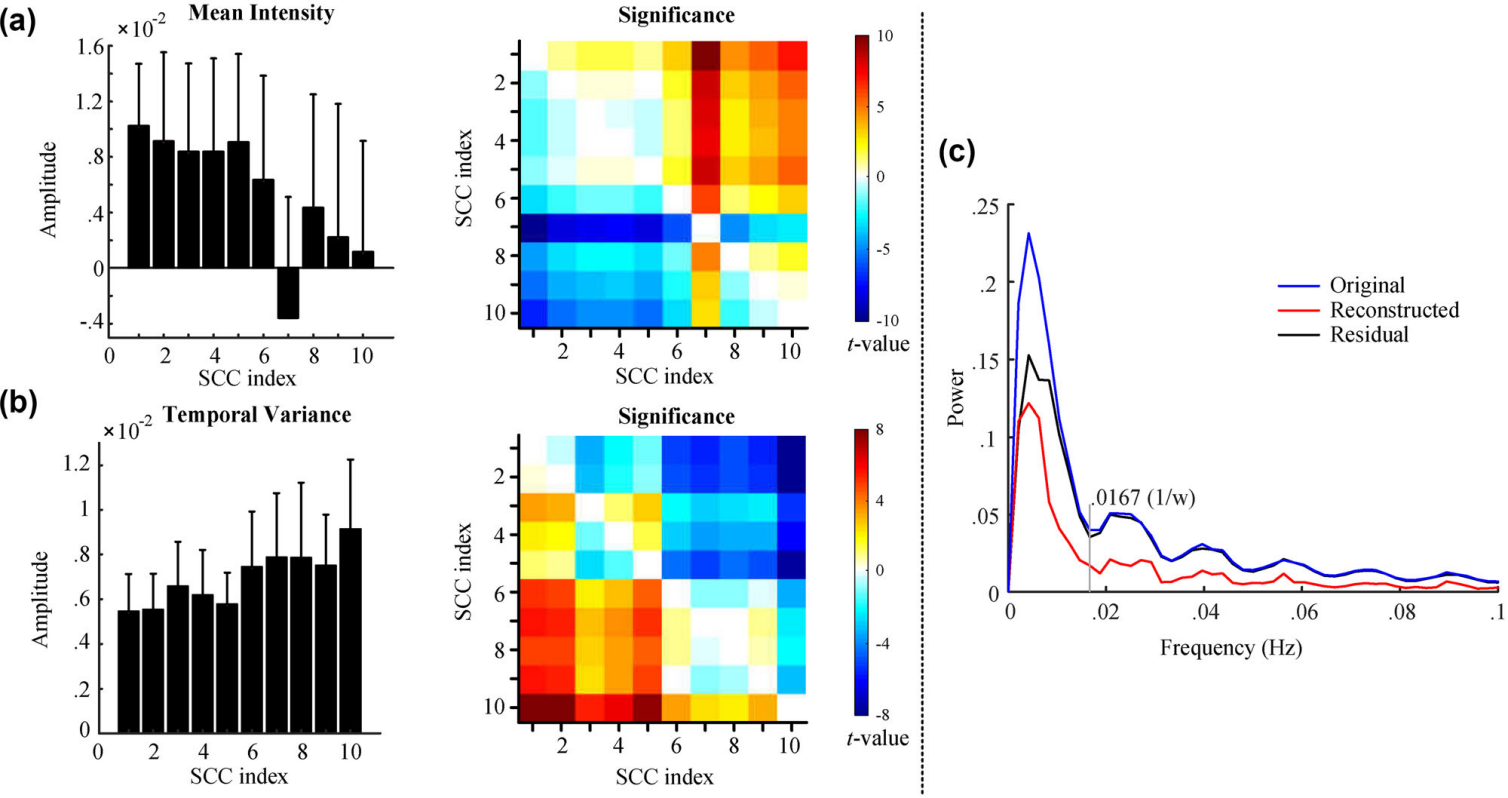
(a and b) The statistical properties of the temporal expression of the 10 group‐level sparse connectivity components (SCCs). (c) The median frequency spectra of the original, reconstructed, and residual correlation time series averaged over all connection pairs were compared for an exemplar subject

We demonstrated that the resting‐state network dynamics were linearly disentangled into a time‐varying combination of SCCs with component‐specific temporal characterization. For the group‐level SCCs (Figure 2c), we calculated the mean intensity and temporal variance of the SCCs (left panel in Figure 4a,b). We observed differentiated temporal properties of these SCCs, with the first SCC possessing the highest mean intensity and minimal temporal fluctuation. The low‐index SCCs, especially SCC 1, had relatively small variances across subjects in terms of mean intensity and temporal variance of time courses. This result indicates that SCC 1 has higher inter‐individual consistency in the statistics of temporal evolution. When the SCC index increased, higher variances in terms of mean intensity and temporal variance of time courses suggested an increased inter‐individual difference in the extent to which the SCC contributes to functional network dynamics. This observation was also supported by the group‐level contrast of mean intensity and temporal variance across the 10 SCCs (two‐sample *t*‐test, *p* < .05) in which we observed significantly higher intensity and lower temporal variability of SCC 1 relative to other SCCs (right panel in Figure 4a,b).

### 3.4 Reconstruction of dynamic functional connectivity

We further explored the potential effects of this reconstruction from a frequency‐domain view. Figure 4c plots the median frequency spectra of the original, reconstructed, and residual correlation time series averaged over all connection pairs for an exemplar subject. A substantial decrease of high‐frequency (> $\frac{1}{\omega }$ Hz) spectral power was observed in the reconstructed correlation time series in contrast to the original time series. Given that the meaningful components concentrate in the frequency interval $\left[0,\frac{1}{\omega }\right]$ Hz due to the low‐pass filtering effects of the sliding window on dFC (Leonardi & Van De Ville, 2015), our findings revealed a significant de‐noise effect of SCC‐based reconstruction on dFC estimation.

### 3.5 Significant differences in the temporal expression of SCCs and classification performance

**FIGURE 5:**
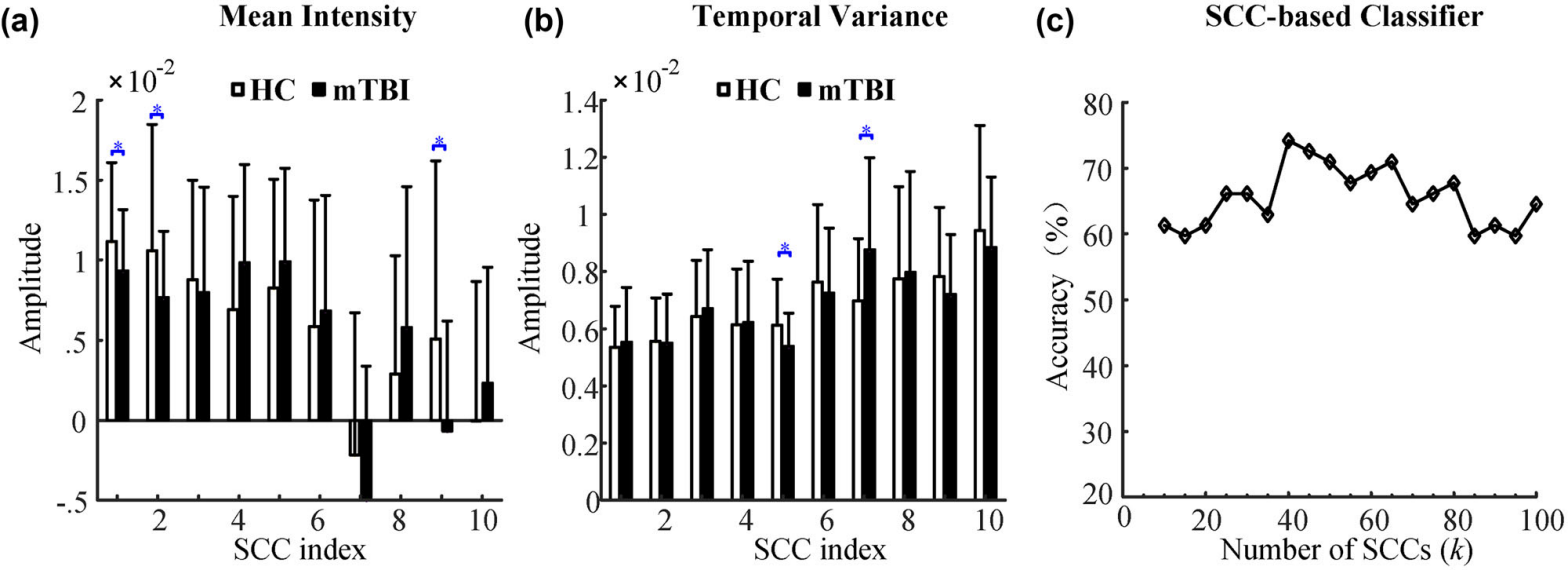
The temporal expression of sparse connectivity components (SCCs) differentiated mild traumatic brain injury (mTBI) patients from healthy controls (HCs). Significant changes in the mean intensity (a) and temporal variance (b) of the SCC time courses (* two‐sample *t*‐test, *p* < .05) are observed. (c) The temporal expression of SCCs exhibited high classification performance using increasing numbers of SCCs *k*

**FIGURE A1:**
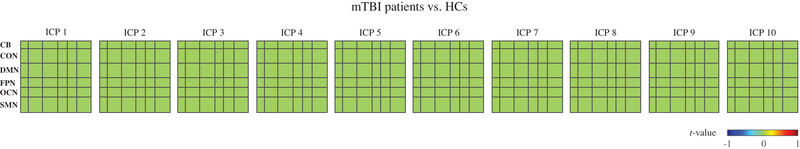
A random effect analysis of a two‐sample *t*‐test was performed on the mild traumatic brain injury (mTBI) patients and healthy controls (HCs) to test the significance of between‐group differences in sparse connectivity components (SCCs). Intriguingly, few significant differences of connection strength in SCCs were found between the mTBI patients and the HCs (*p* < .05, false discovery rate (FDR)‐corrected)

Intriguingly, we found few significant differences in the connection strength of SCCs between patients with mTBI and HCs (see Figure A1). However, significant changes (two‐sample *t*‐test, *p* < .05) in the mean intensity (Figure 5a) and temporal variance (Figure 5b) of the SCC time courses were detected in resting‐state scans in mTBI patients compared with HCs, involving SCC 1, SCC 2, SCC5, SCC 7, and SCC 9. As shown in Figure 2c, these SCCs were related to multiple brain networks such as DMN, CB, FPN, and SMN, indicating that the interactions of functional networks were more likely to be damaged in mTBI patients compared with HCs. We further tested whether the temporal expression of SCCs could discriminate mTBI patients from HCs. Figure 5c shows the classification accuracies using different numbers of SCCs *k*. The SCC‐based classifier achieved an optimal classification accuracy of 74.2% (64.5% for HCs, 83.9% for mTBI) with k=40. The two‐sample *t*‐test results of behavior performances between HCs and mTBI patients are provided in Table 2. We found a significant difference between mTBI patients and HCs in only the digit symbol task (*p* < .05). However, we found no significant correlation between mean intensity or temporal variance of the SCCs and digit symbol task. Overall, the SCC‐based classifier showed a high classification performance, suggesting its potential in assisted diagnosis of mTBI.

### 3.6 Reproducibility and reliability of SCCs

**FIGURE 6:**
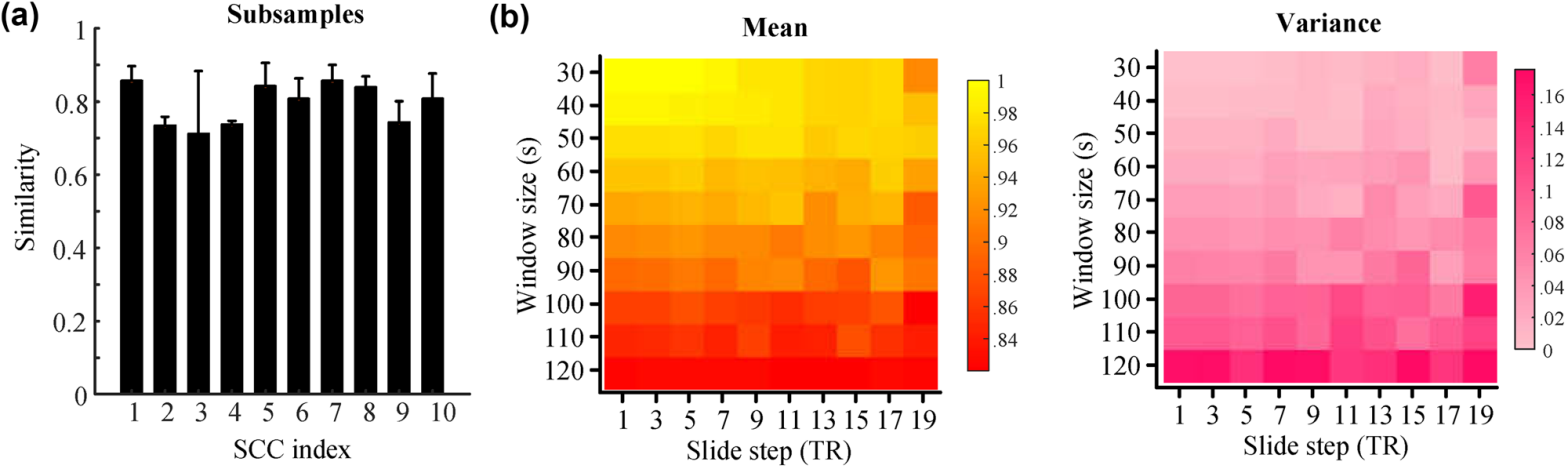
Sparse connectivity components (SCCs) exhibited great reproducibility and reliability. (a) The 10 SCCs had high spatial similarity with the SCCs on three subsamples of the dataset. (b) High mean and low variance of the similarities of correlation maps were observed across the 10 SCCs compared with the baseline in different combinations of window size and step size, demonstrating high reproducibility of SCCs with varying sliding‐window parameters

The reproducibility and reliability of SCCs in various conditions were further assessed. Notably, the obtained SCCs had high spatial similarity to the SCCs on three subsamples of the dataset ($\eta^{2}=\;0.79\pm 0.08$; Figure 6a). In addition, we observed higher means and lower variances in the similarity of SCCs for smaller window sizes (Figure 6b). Compared to the window size, the sliding step has less impact on the SCCs. All of these results demonstrated the high reproducibility of SCCs with varying sliding‐window parameters.

## 4 DISCUSSION

In our study, the temporal evolution of functional brain networks was delineated by time‐varying combinations of a set of overlapping SCCs with sparse dictionary learning based on dFC. Then, using the subject‐specific temporal expression of these SCCs, we achieved a classification accuracy of 74.2% between mTBI patients and HCs (64.5% for HCs, 83.9% for mTBI patients).

The identified SCCs exhibited meaningful spatiotemporal configurations, which extends our understanding of spontaneous fluctuation in resting‐state FC. SCC 1, which had the maximal norm, exhibited the greatest importance in the temporal expression of functional network dynamics. This result was supported by converging evidence. For example, SCC 1 had the maximal mean intensity as well as the minimal variance in the corresponding time courses among the 10 SCCs. SCC 1 was expressed more consistently across subjects, suggesting its overwhelming stability across subjects as a source in network dynamics. More importantly, SCC 1, which is spatially characterized by a clear separation of six functional brain networks with strong intra‐network connectivity, showed a highly similar connectivity profile to the static connectivity maps. Taken together, these specific spatiotemporal properties suggest that SCC 1 may play a baseline role in functional network dynamics. Those high‐index SCCs with small mean expression intensity and great temporal variability possess significant between‐network connectivity patterns, indicating that functional network dynamics are predominantly situated across networks (Shen et al., 2016). This architecture of functional coupling reflects the nature of cognitive information processing via dynamic interplay across different functional subsystems in the brain (Power et al., 2013; Zalesky et al., 2014). Finally, while we are unable to explicitly link these SCCs with specific cognition functions in this study, some of the identified connectivity configurations within individual SCCs likely imply the underlying biological meaning. For instance, the well‐known anticorrelation between the DMN and task‐positive networks was evident in both SCC 5 and SCC 7, suggesting these two components respond to the task‐negative or task‐positive brain states, respectively. Some focal between‐network couplings were separately identified by some SCCs, such as the DMN‐FPN interaction in SCC 8 and the SMN‐DMN connection in SCC 7.

**FIGURE 7:**
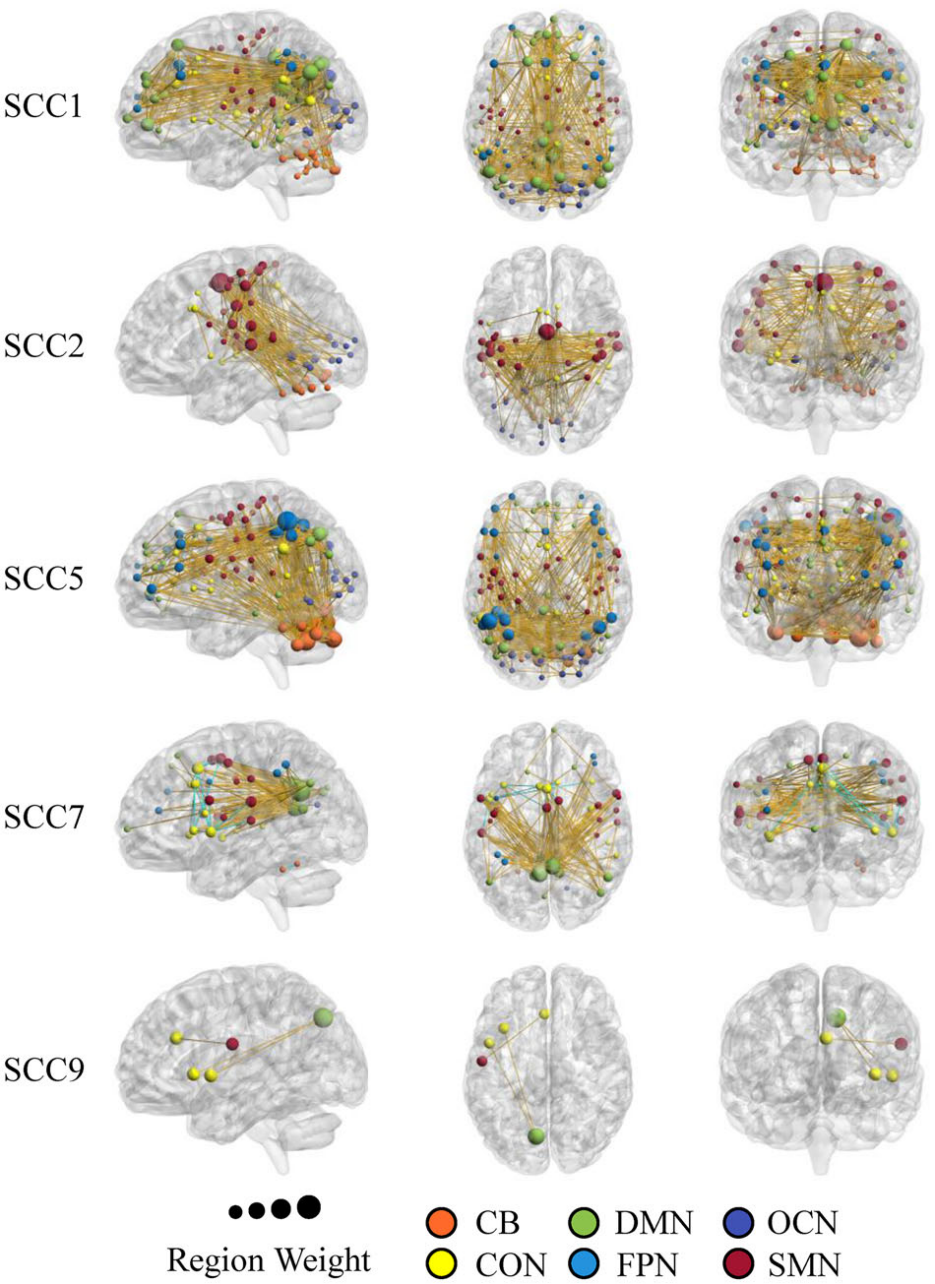
Connectivity patterns of five significant different sparse connectivity components (SCCs) between the mild traumatic brain injury (mTBI) patients and healthy controls (HCs). The size of nodes indicates the amount of connectivity of the regions of interest (ROIs). Different colors of the nodes indicate different brain regions. The yellow and bule lines represent significant positive and negative correlations, respectively

Significant changes in the mean intensity and temporal variance of some SCCs’ time courses were detected in the patients with mTBI compared with the HCs, especially that of SCC 1, SCC 2, SCC 5, SCC 7, and SCC 9. The connections with high *t*‐values (|t|> 6.0) of these SCCs are depicted in Figure 7. SCC 1 shows a highly similar connectivity profile to the static connectivity maps, indicating that mTBI may strongly affect static functional connectivity between various brain regions. Similarly, it was reported that patients with mTBI show greater static functional connectivity in cingulate, temporal, and frontal regions than HCs (Tang et al., 2011). Furthermore, there were significant differences in SCCs related to several networks, which is consistent with previous studies (Johnson et al., 2012; Kasahara et al., 2010; Mayer et al., 2011; Palacios et al., 2017; Sours et al., 2013; Vakhtin et al., 2013; Vergara et al., 2017; Zhou et al., 2012). Specifically, decreased functional connectivity was found in the DMN and frontal cortex, whereas the visual network showed increased functional connectivity (Mayer et al., 2011; Palacios et al., 2017). It was also shown that abnormal functional connectivity alterations in the DMN mainly existed in the semiacute phase of injury (Johnson et al., 2012; Palacios et al., 2017; Sours et al., 2013; Zhou et al., 2012). Early studies also reported alteration of functional connectivity in sensorimotor areas (Kasahara et al., 2010; Vakhtin et al., 2013). Moreover, significant abnormal connections within the cerebellum have been observed, which was considered an important region for mTBI (Nathan et al., 2015; Vergara et al., 2017). Connectivity disruption of other areas was also reported including the hippocampal, primary visual, dorsolateral prefrontal cortexes (S. Slobounov et al., 2011), and the thalamus (Tang et al., 2011; Zhou et al., 2014).

The findings of functional connectivity alterations are significant and important to achieve applications in clinical settings. The changes in functional coupling across regions could serve as a biomarker in the diagnosis of mTBI and may aid clinicians in diagnosis. For example, Kim et al. (2013) reported an optimal classification accuracy of about 71% in discriminating mTBI in the semiacute phase of injury and healthy brains with diffusion tensor imaging (DTI). Functional connectivity has also been used for discriminating mTBI patients in the semiacute injury stage and achieved an accuracy of 84.3% (Mayer et al., 2011). However, these previous studies were based on the semiacute stage, and individuals may not show obvious structural changes or symptoms during the early acute stage (Mayer et al., 2010; Milman et al., 2005). Thus, the evaluation of mTBI in the early acute stage is more challenging. Our findings demonstrated that the temporal expression of SCCs could achieve a high classification performance in classifying mTBI patients in the early acute phase, suggesting that the changes in dynamic functional coupling across regions serve as neurobiological biomarkers in the diagnosis of mTBI.

From behavioral data, we found that the significant difference between mTBI patients and HCs existed only in the digit symbol task (*p *< .05). This may be attributed to the fact that most of the mTBI patients may have unapparent symptoms in the early acute stage (Milman et al., 2005). However, at the semiacute stage or later, patients with mTBI start to exhibit neurological symptoms and cognitive defects (Madhavan et al., 2019; McAllister et al., 2006; R. Ruff, 2005). Some patients will even develop persistent neurological symptoms and cognitive defects after 1 year.

Some limitations should be mentioned for the present study. The small sample size of subjects and lack of longitudinal clinical data are major issues limited by the rareness of available patients with mTBI. Prior studies have reported the longitudinal correlation between white matter microstructural changes and neuropsychological performance (Palacios et al., 2020). Therefore, in future work, we will include longitudinal data to better explore potential associations between imaging abnormalities and behavior changes. Another potential limitation is the use of a 15‐channel head coil for data acquisition. The MRI scanner with 32‐channel head coil could provide higher signal‐to‐noise ratio and sensitivity fMRI data (Kaza et al., 2011), which would be beneficial to capture more subtle functional alterations in mTBI. However, the 15‐channel head coil is widely used in clinical settings, and therefore may be more applicable in those settings.

## 5 CONCLUSIONS

In conclusion, we applied a methodology built on sparse dictionary learning of windowed correlation to delineate subject‐specific time‐varying evolution using a set of overlapping group‐level SCCs. The identified SCCs with hierarchical spatial structures were consistently distributed in the cohort of subjects without significant differences between patients with mTBI and HCs. Further, the observed temporal expression profiles of the SCCs were predictive of mTBI, which may be helpful for the individualized diagnosis of this disease. These findings suggest that the time‐varying combinations rather than the coupling profiles of the SCCs are essential for revealing the pathologic basis of mTBI.

## CONFLICT OF INTEREST

The authors declare no conflict of interest.

## AUTHOR CONTRIBUTIONS

Hui Shen designed the experiments. Liangwei Fan and Huaze Xu performed the experiments. Liangwei Fan, Jian Qin, Kai Gao, and Min Ou interpreted the results of the experiments. Liangwei Fan, Hui Shen, and Jianpo Su wrote the manuscript. Na Li participated in the fMRI data acquisition. Song Peng participated in the behavior data acquisition. All authors have read and agreed to the published version of the manuscript.

### PEER REVIEW

The peer review history for this article is available at https://publons.com/publon/10.1002/brb3.2414

## Data Availability

The data that support the findings of this study are available on request from the corresponding author. The data are not publicly available due to privacy or ethical restrictions.
